# Charles A. Meeker, D.D.S.

**Published:** 1913-10-15

**Authors:** 


### CHARLES A. MEEKER, D.D.S.

In the death of Dr. Charles A. Meeker, September 8th, 1913, at his home in Newark, N. J., the dental profession looses another of its good and faithful servants. Dr. Meeker was born 1846. He graduated from the Baltimore College of Dental Surgery in 1884, and has been in active practice and professional work for a much longer time. He was Secretary of the New Jersey State Dental Society for 26 years. He was for many years a member of the New Jersey Board of Dental Examiners, and for 16 years Secretary and Treasurer of the National Association of Dental Examiners. He has all his life been a most active participant in national and home professional affairs. He was a man of strong attachments to friends. He had a unique personality which drew most men to him, but unfortunately repelled some. He was wholly devoted to his profession, and was ever ready to give his time or services to any cause which would promote fraternal or professional good. He was widely known in the profession for his activities in professional affairs. He was never a large contributor to our professional literature. At the time of his death he was and had been since its establishment editor of The Dental Scrap Book, which is characteristic of Meeker in that it is one of our most spicy and valued magazines.

Dr. Meeker will be missed at our National gatherings and he surely will be missed in the New Jersey and New York meetings which he always attended and made interesting by his active participation. His death was unexpected and surely untimely, as he should have had many years yet of activity.

